# Perceived family support regarding condom use and condom use among secondary school female students in Limbe urban city of Cameroon

**DOI:** 10.1186/1471-2458-14-173

**Published:** 2014-02-18

**Authors:** Elvis E Tarkang

**Affiliations:** 1 HIV/AIDS Prevention Research Network, Cameroon (HIVPREC), Opposite Premier Pharmacy, Commonwealth Avenue, P.O Box 36, Kumba, Cameroon

## Abstract

**Background:**

HIV/AIDS prevention programs rooted in the social cognitive models are based on the theoretical assumptions that adoption of preventive behaviour (condom use) depends on the individual’s perceptions of their susceptibility to HIV/AIDS and the benefits of condom use. However some studies contend that applying such models in the African setting may not be that simple considering that in many societies, people’s capacity to initiate health enhancing behaviour are mediated by power relations (parents/guardians) and socialisation processes that are beyond the control of individuals. The relative influence of these family forces on condom use is however unknown in Cameroon. In this study it is hypothesized that adolescents’ perceptions of family support for condom use, would encourage condom use among female students in Limbe urban city of Cameroon.

**Methods:**

A cross-sectional study of a probability sample of 210 female students selected from three participating secondary school was adopted, using a self-administered questionnaire to collect data. Pearson Chi-square statistics was used to test association between perceived family support for condom use and condom use. Statistics were calculated using SPSS version 20 software program.

**Results:**

Of the respondents, 56.2% reported being sexually active. Of these, 27.4% reported using condoms consistently; 39.1% reported having used condoms during their first sexual intercourse, while 48.7% reported having used condoms during their last sexual intercourse. Majority of the female students exhibited positive perceptions regarding family support for condom use. Respondents who agreed that they feel themselves free to discuss condom use with their parents or any adult member of the family, reported more condom use during first sex than those who disagreed (X^2^ = 13.021; df = 6; p = 0.043). Likewise respondents who agreed that they feel themselves free to discuss condom use with their parents or any adult member of the family, reported more condom use at least once, than those who disagreed (X^2^ = 8.755; df = 3; p = 0.033).

**Conclusion:**

Significant associations between perceptions of family support for condom use and condom use were established in this study. This finding suggests that regardless of the sexual communication patterns within the family, techniques that increase the occurrence of parent and female student’s discussion concerning condoms and HIV/AIDS will prove useful in preventing HIV/AIDS among female students in Limbe Urban City of Cameroon.

## Background

Concern about young people’s vulnerability to new HIV infections has led to a deluge of youth-oriented reproductive health programs focusing on protective behaviour especially condom promotion [1], as a means of stemming the tide of infection. The theoretical assumption of these programs (Health Belief Model, Social Cognitive Theory, Theory of Reasoned Action, AIDS Risk Reduction Model and Stages of Change Model) is that the adoption of productive behaviour is based on an individual’s perceptions of their susceptibility to infections and the benefits of behaviour change. However these models include a variety of constructs and they do not rely solely on individual aspects as determinants of behaviours. People are seen as rational beings who logically consider various courses of action before acting, once they have adequate information and see the benefits of change. However, some studies contend that applying such theories in the African setting may not be that simple considering that in many societies, people’s capacity to initiate health enhancing behaviour are mediated by power relations, poverty gender inequality and socialization processes that are beyond the control of individuals [2].

Existing research also suggests that the social environment of adolescents is an influential factor in the decision to use condoms [3-5]. Individuals form their own views in consonance with or in opposition to the dominant norms of their peers, family and society. Some studies [6], found that parental support was a significant predictor of condom use among adolescents in urban Cameroon, especially young women. Family support is therefore critical, given the strong influences that the family environment exerts on adolescent sexuality especially in developing societies. The relative influence of these family forces on condom use is however unknown in the Southwest region of Cameroon.

Sexual risk behaviour among adolescents is a major public health problem. People under 25 comprise approximately half of all new HIV cases, the majority of whom are infected through unprotected sex [7,8]. Condom use is effective in preventing HIV infection and some family-based prevention programs hope to increase safer sex by improving parent-adolescent communication [9]. Thus understanding more about the relationship between condom use and perceived parent-adolescent communication regarding HIV/AIDS and condom will likely have important implications for HIV prevention for adolescents especially girls.

Several studies of community based student samples suggest that when parents talk with their teens about sex, adolescents report greater contraceptive use, including condom use [10,11]. For example mother-child discussion about condom use prior to sexual debut has been correlated with adolescent condom use at last sex [12]. Likewise, a similar study found that 76% of sexually active adolescents, who reported having had a conversation with either parent about condoms, used a condom at most recent intercourse and also reported greater lifetime condom use than those who had not discussed condoms with a parent [13].

In contrast, other studies have linked parent-teen communication with increased sexual risk behaviours such as unprotected sexual intercourse [14], or have found no relationship at all [15,16]. Some studies have shown that adolescent rather than parental perceptions of communication are more predictive of adolescents’ sexual behaviour [17].

Sex education has rarely been a comfortable topic for parent-child communication. Many parents are unwilling to talk about sex or uncomfortable doing so, or they may lack the knowledge themselves. Many barriers might prevent open communication between parents and children about sexual issues. For example, adults fear that informing adolescents about sex and teaching them how to protect themselves will make them sexually active [18]. Parents play a passive role in providing information to their children, yet they are expected to be key players in this role. This is because sex in most African societies is a taboo subject between parents and children. Parents fail to fulfil their role of educating their children on sex, sexuality and HIV/AIDS [19]. This is reflected in the high prevalence rate of HIV/AIDS among female adolescents of reproductive age in Cameroon, 6.8% [20].

Previous studies on parent-adolescent communication about sexual issues mostly in the United States of America (USA) have shown some associations with condom use [10-13]. However the process of parent-adolescent communication is different from the individual perceptions of family support for condom use. To date no study has examined the effect of adolescent perception regarding family support for condom use on condom use among adolescent in Cameroon. The current study aimed to examine the association between perception of family support for condom use and condom use among high school female students in Limbe Urban city of Cameroon.

It was hypothesized that adolescents’ perceptions of having specific HIV/AIDS and condom discussions and perceptions of open sexual communication would encourage condom use among female students in Limbe, Cameroon.

## Methods

A cross sectional research design was adopted to examine the perception of family support for condom use among high school female students in Limbe urban area of Cameroon, collecting data through self-administered questionnaires.

The questionnaire was pretested on 10 students who did not take part in the actual study, to clarify instructions, relevancy, usability and completion time, to refine and introduce modifications where necessary and to ascertain reliability and validity [21]. The reliability of the research instrument used for the study was tested using the coefficient alpha, and by pre-testing the questionnaires. The following types of validity were also established: face validity, content validity, construct validity and criterion-related validity. This was ensured by constructing items to represent the different components of the study, based on literature review.

The questionnaires were distributed to a stratified simple random sample of 210 female students in three high schools in Limbe urban area of Cameroon during normal class periods with the permission of the principals and the co-operation of the teachers concerned. One research assistant was available to assist the students and to answer questions while they completed the questionnaires during a classroom period. Probability sampling was used because it increased the likelihood that all the elements in the population would have an equal chance of being included in the sample [22]. The school attendance registers of the students were used as the sampling frame to select a sample of 210 grade 10 to grade 12 (Form five to upper sixth) female students randomly from three senior secondary schools in Limbe urban area of Cameroon. A list of students registered in high school level in the participating school was obtained. The sample frame consisted of a consecutively numbered name list of the respondents in the three participating high schools. The respondents were stratified at different levels of study, namely grade 10, grade 11 and grade 12. After stratification, a simple random sample was obtained by placing all the numbers corresponding to the name list in a container and selecting the stipulated sample size. There are about 20 high schools in Limbe urban area. All the principals were contacted and permission to carry out the study was sought. Only three principals granted permission and due to time, financial and logistical constraints, the researcher had to go ahead and collect data from these three schools. These three schools were thus conveniently chosen by the researcher.

Data collection took place in October of 2012. The sample size of this study was determined using the formula for a single population proportion [23]. Permission to conduct this study was granted by the HIV/AIDS Prevention Research Network, Cameroon (HIVPREC), a Non-governmental Organisation (NGO) for the prevention of HIV/AIDS through formalized education, working in the South West region of Cameroon, and the principals of the three participating high schools. Participation was voluntary and informed written consent was obtained from each student and her parent/guardian prior to data collection. A questionnaire was handed to a student when she produced the signed consent form from a parent/guardian and from herself.

Anonymously completed questionnaires were kept in a separate container from the signed informed consent forms in order to maintain anonymity. Anonymity was also maintained by reporting the findings of the three schools combined and by not providing comparisons among the three schools. Confidentiality was maintained because only the researcher had access to the completed questionnaires, which were locked up. Subsequent to the acceptance of the research report, these would be destroyed. Data were collected in three sections: socio-demographic characteristics, perceptions regarding parent-adolescent communication on HIV/AIDS and condom and condom use. Data for this study was part of the data set of a bigger study that investigated the knowledge of HIV/AIDS and sexual behaviours among female students in Limbe in the Southwest region of Cameroon.

### Data analysis

Data was analysed using SPSS version 20 software program. Data was summarized by means of descriptive statistics including the frequency table. More advanced statistics included the chi square test at the 0.05 significant level to test the association between perception of family support for condom use and condom use.

### Measures

• ***Sexual experience***

 This was measured with the question: Have you ever had sexual intercourse with a male partner? With ‘1 = yes’ or ‘0 = no’ as response options.

• ***Perceived benefit of condom use***

 This measure was based on the degree of agreement with the following statement: ‘Correct and consistent use of condoms during sexual intercourse could prevent transmission of HIV/AIDS’. The response options were ‘3 = strongly agree’, ‘2 = agree’, ‘1 = disagree’, and ‘0 = strongly disagree’.

• ***Condom use during first sexual intercourse***

 This measure was derived from the question: “Did you use a condom the first time you had sexual intercourse with a male partner?” The response option was dichotomized as ‘1 = yes’ or ‘0 = no’. This question was asked only to respondents who had experienced sex.

• ***Condom use during last sexual intercourse***

 This measure was derived from the question: “Did you use condom the last time you had sexual intercourse with a male partner?” The response options were ‘1 = yes’ or ‘0 = no’. This question was also asked only to respondents who are sexually active.

• ***Ever used condoms***

 This measure was derived from the question: “Have you ever used a condom during sex?” The response options were ‘1 = yes’ or ‘0 = no’. This question was asked to all the respondents.

• ***Consistent condom use***

 The outcome variable for this study is consistent condom use during sexual intercourse as reported by the female learners. This measure was derived from the question:

•“How often do you use a condom with a partner during sexual intercourse?” The response options were: ‘1 = always’, ‘2 = most of the time’, ‘3 = seldom’ and ‘4 = never.’ This question was asked only to respondents who were sexually active.

• ***Perceived family support for condom use***

 This measure was assessed by asking the respondents to state their degree of agreement with the following seven statements considered separately: “I feel myself free to discuss condom use with my parents or any adult member of the family”; “I feel free to discuss HIV/AIDS with my parent or guardians”; “parents are supposed to talk sex with their children”; “it is culturally acceptable for parents to discuss sex with their children”; “my parents/guardians are knowledgeable about HIV/AIDS”; “my parents/guardians are knowledgeable about condom use”; and “my parents/guardians support condom use”. The Cronbach’s alpha for this 7-item scale was 0.736. The response options were rated on a four-point Likert scale as ‘3 = strongly agree’, ‘2 = agree’, ‘1 = disagree’ and ‘0 = strongly disagree’. ‘Strongly agree’ and ‘agree’ were coded as the index category.

• ***Socio-demographic variables***

 The following socio-demographic variables were included in the study: age group, marital status, and religious affiliation. Age was self-reported by respondents in years. Marital status was dichotomized as ‘single’ (index category) and ‘married or cohabiting’. Religious affiliation was dichotomized as ‘Christian’ (index category) and ‘others’.

## Results

### Descriptive statistics

Most students, 92.4% were 16-24 years old. Most, 92.3% were single and all were secondary school female students. Of the respondents, 94.7% were Christians.

Regarding perceived family support for condom use, a slight majority of the respondents, 50.5% perceived that they feel free to discuss condom use with their parents or any adult member of the family; a great majority, 66.1% perceived that they feel free to discuss HIV/AIDS with their parents or guardians; 69.3% perceived that parents are supposed to talk sex with their children; 65.6% perceived that it is culturally acceptable for parents to discuss sex with their children; 82.5% perceived that their parents/guardians are knowledgeable about HIV/AIDS; 81.7% perceived that their parents/guardians are knowledgeable about condom use; and 65.9% perceived that their parents/guardians support condom use (Table 1).

**Table 1:** Perceived family support for condom use of secondary school female students

**Perceived family support for condom use**	**Frequency**	**Percentage**
I feel myself free to discuss condom use with my parents or any adult member of the family (n = 180)		
*Agree*	91	50.5
*Disagree*	89	49.5
I feel free to discuss HIV/AIDS with my parents or guardians (n = 180)		
*Agree*	119	66.1
*Disagree*	61	33.9
Parents are supposed to talk sex with their children (n = 176)		
*Agree*	122	69.3
*Disagree*	54	30.7
It is culturally acceptable for parents to discuss sex with their children (n = 180)		
*Agree*	118	65.6
*Disagree*	62	34.4
My parent/guardians are knowledge about HIV/AIDS (n = 175)		
*Agree*	146	82.5
*Disagree*	29	17.5
My parents/guardians are knowledgeable about condom use (n = 173)		
*Agree*	143	81.7
*Disagree*	30	18.3
My parents/guardians support condom use (n = 173)		
*Agree*	114	65.9
*Disagree*	59	34.1

The perceived benefit of using condoms to prevent HIV/AIDS was quite high. Most of the respondents 79.5% agreed that correct and consistent condom use can prevent HIV/AIDS. The majority, 56.2% had experienced sexual intercourse. Of the sexually experienced respondents, 39.1% used condoms during their first sexual encounters, 48.7% used condoms during their last sexual encounters and 48.9% had used condoms at least once. Only 27.4% were using condoms consistently (Table 2).

**Table 2:** Condom use among secondary school female students in Limbe urban city

**Characteristics**	**Frequency**	**Percentage**
**Sexual experience (n = 201)**		
- Yes	113	56.2
- No	88	43.8
**Perceived benefit of condom use (n = 195)**		
- Correct and consistent condom use can prevent HIV/AIDS		
*Agree*	155	79.5
*Disagree*	40	20.5
**Regularity of condom use (n = 113)**		
- Always	31	27.4
- Most of the time	34	30.1
- Seldom	19	16.8
- Never	29	25.7
**Condom use during first sexual encounter (n = 110)**		
- Yes	43	39.1
- No	67	60.9
**Condom use during last sexual encounter (n = 113)**		
**-** Yes	55	48.7
- No	58	51.3
**Ever used condoms during sexual encounter (n = 180)**		
**-** Yes	88	48.9
- No	92	51.1

### Associations between perception regarding family support for condom use and condom use

The study reveals the following significant associations between perception regarding family support for condom use and condom use:

Respondents who agreed that they feel themselves free to discuss condom use with their parents or any adult member of their family (58.3%) were more likely to have ever used condoms than those who disagreed, (38.0%) (X^2^ = 8.755; df = 3; p = 0.033).

Respondents who agreed that they feel themselves free to discuss condom use with their parents or any adult member of their family, (44.8%) were more likely to have used condoms during their first sexual encounters than those who disagreed, (X^2^ = 13.021; df = 6; p = 0.043).

## Discussion

The majority of the respondents were among the age group hardest hit by HIV/AIDS [24]. Vulnerability to HIV/AIDS (especially among youth) relates to non-consistent condom use and the impossibility to negotiate its use [25].

Gender inequality places women at a greater risk of being infected by HIV/AIDS. Women and young girls lack power over their bodies, and their sexual lives, social and economic inequalities increase their vulnerability for contracting and living with HIV/AIDS. With increasing levels of poverty in Cameroon, women especially female students have found themselves in casual relationships with men for financial gains. Women might therefore find it difficult to demand condom use, as they become subordinates or dependent of mainly older men; women are also biologically prone to infection, and HIV is more easily transmitted from men to women than the reverse [26].

Religion could hamper the effective use of condoms for HIV prevention [27]. The Roman Catholic Church opposes condom use in favour of “direct contact” [28,29]. This could have serious implications for spreading HIV. Religious beliefs could significantly shape individual’s use of condoms. Since its very inception, the Roman Catholic Church has forbidden the application of contraceptive measures because it considers that such interference is a transgression of divine law and a sin against nature. The use of contraceptive is prohibited whatever the circumstances. This also applies to the condom even when one intends to use it, not to prevent pregnancy, but to avoid fatal infections such as HIV/AIDS. For instance, the use of a condom within the marital relationship even when one of the spouses is HIV positive is considered sinful. This interdict has had the direct consequence with regard to the implementation of AIDS prevention programmes among Christians, especially Catholics in the developing countries. The protestant churches also emphasise birth control basing their views on their puritan tradition and responsibility founded on a Christian social ethics.

This study provides important information concerning perception of parent-adolescent discussion of sexual topics. Female students in Limbe urban city of Cameroon reported positive perceptions regarding parent-adolescent communication on HIV/AIDS and condom.

Majority of the respondents perceived that parents-adolescents discussion on HIV/AIDS and condom was necessary. Since majority of these female respondents, 56.2% were sexually experienced, such perceptions regarding family-adolescent discussion on HIV/AIDS and condoms were certainly appropriate, given the frequent rate of high risk behaviour among the female students (unprotected sexual intercourse) [30,31].

Importantly, in accordance with other studies, and in the context of HIV/AIDS [4,32], this study demonstrates significant associations between perceived family support for condom use, and condom use, with students who felt themselves free to discuss condom use with their parents or any adult member of their families being more likely to use condoms during sexual intercourse. Therefore facilitating family support for condom use among sexually active girls is important toward empowering them to adopt protective behaviour.

The finding suggests that the perceived family support for condom use was an important factor influencing condom use. Thus the general openness of the perceived family support for condom use was related to the female student’s safe sexual behaviour (condom use).

This finding suggests that regardless of the sexual communication patterns within the family, techniques that increase the occurrence of parent and female student’s discussion concerning condoms and HIV/AIDS will prove useful in preventing HIV/AIDS among female students in Limbe Urban City of Cameroon.

The social environment is an important determinant of sexual behaviour among young people. Thus perceptions of support for adolescent condom use from significant others in the community (especially the elderly) can considerable influence their actions. The proportion of respondents who perceived support for condom use from parents and other adult in the family was high. Such perceptions may encourage girls to be more enthusiastic and able to use condoms since they are more affected by the negative consequences of sexual activity.

Some studies focused on the possible influences of parents on HIV and condom use among adolescents. Literature shows that, the relative connectedness of adolescents to significant others may be important protective factors, and thus operate as suppressors of risk. In families where reproductive health issues can be discussed freely and where condom use is condoned or approved of, individuals are more likely to actually use them [5,33]. In societies where the family is still an important principle of social organization and where social organization tends to be hierarchical such as in Cameroon, the opinion of family members or community leaders is likely to matter most. In many African societies, parents, family and community members still feature prominently in the everyday life of the adolescents. Because adolescents still depend on them financially, emotionally, socially as well as culturally, these actors continue to influence adolescent behaviour through (anticipated) positive and negative sanctions. Family and community structures can remain important frames of reference and therefore remain relevant contexts for decisions concerning reproductive health behaviour. Furthermore, opinion leaders have a dual function; they not only disseminate information, but also express their approval/disapproval of various behaviours.

Although some reporters have documented limited communication about sexuality issues between adults and young people [34], the results of the study highlight the importance of encouraging communication about sexual health between adults and young people as this is likely to have an effect on perceptions of family support and therefore encourage the adoption of protective behaviour among young people.

Availability is not an obstacle to condom use since cheap condoms are readily available in Cameroon. Therefore the low prevalence of consistent condom use by female students in this study, 27.4%, may be the result of factors, including underestimation of risk of HIV and the constraints of the social environment such as lack of family support for condom use. Individuals may not be able to protect themselves even if they want to because of socio-cultural constraints. Young women are particularly at risk in this regard as the combined effects of gender inequality and family constraints may dis-empower them, thereby increasing their vulnerability.

This study should be interpreted in the light of some limitations. These include possible bias in reporting condom use among the female students. However, assurance of confidentiality and anonymity might have minimised this problem. The sample size was small and the sample was homogeneous. The lack of more sophisticated statistical analysis might also limit the study. Also, collection of data from one geographical location and from an urban area may limit the ability to generalise the study results.

Notwithstanding these limitations, the results of this study suggest that efforts to promote open communication between parents and adolescents regarding HIV/AIDS, condom use and sexuality, should be encouraged and augmented, as this will increase condom use among female adolescents.

Although condom use among youths is currently being promoted in Cameroon, little attempt is being made to encourage family support for adolescent condom use. Programme planners should implement strategies to supress barriers to parent-adolescent communication regarding sexuality, HIV/AIDS and condom use.

## Conclusion

This study found evidence of association between perceptions regarding family support for condom use and condom use among female students in Limbe urban city of Cameroon. The hypothesis that adolescents’ perceptions of having specific HIV/AIDS and condom discussions and perceptions of open sexual communication would encourage condom use among female students in Limbe, Cameroon, was accepted at the 0.05 level of significance.

### Recommendation

Since this study was conducted on female students from predominantly Christian area of Cameroon, a similar study should be conducted in predominantly Muslim area of Cameroon, and also on male students. A qualitative research should also be carried out to ascertain the nature of parent-adolescent communication regarding HIV/AIDS and condom, and their association with condom use.

## Competing interests

The author declares that he has no competing interests.

## Pre-publication history

The pre-publication history for this paper can be accessed here:

http://www.biomedcentral.com/1471-2458/14/173/prepub
